# A Cross-Sectional Study on the Agreement of Perfusion Indexes Measured on Different Fingers by a Portable Pulse Oximeter in Healthy Adults

**DOI:** 10.7759/cureus.24853

**Published:** 2022-05-09

**Authors:** Sharada M Swain, Manju Lata, Sandeep Kumar, Shaikat Mondal, Joshil K Behera, Himel Mondal

**Affiliations:** 1 Department of Physiology, Hi-Tech Medical College and Hospital, Bhubaneswar, IND; 2 Department of Physiology, Employees' State Insurance Corporation Medical College and Hospital, Patna, IND; 3 Department of Physiology, Raiganj Government Medical College and Hospital, Raiganj, IND; 4 Department of Physiology, Saheed Laxman Nayak Medical College and Hospital, Koraput, IND

**Keywords:** oximetry, photoplethysmography, reliability, plethysmography, perfusion index, oxygen saturation, oximeter

## Abstract

Background

Pulse oximeters measure oxygen saturation, heart rate, and perfusion index (PI) by analyzing photoplethysmographic signals. PI is an indirect measure of peripheral perfusion expressed as a percentage of pulsatile signals to non-pulsatile signals. PI measured from different sites may show variation. PI may vary when measured on different fingers. In this study, we aimed to observe the variation of PI among different fingers of both hands.

Methodology

This cross-sectional, observational study was conducted using a convenience sample recruited from a tertiary care hospital in eastern India. PI was measured in apparently healthy adults in a sitting posture after a five-minute rest. The pulse oximeter probe was attached to each finger and readings were taken after one minute. The analysis of variance and intraclass correlation coefficient (ICC) were calculated to compare and find agreement among PI.

Results

Data from a total of 391 (229 [58.57%] male and 162 [41.43%] female) adult research participants with a mean age of 34.88 ± 10.65 years were analyzed. The PI was the highest on the middle finger in both hands. There was a significant difference among the PI measured on different fingers, F (9, 3900) = 15.49, p <0.0001. The ICC was 0.474, 0.368, and 0.635 for overall, right-hand, and left-hand fingers, respectively, which indicate poor (ICC < 0.5) to moderate (ICC = 0.5-0.75) levels of reliability.

Conclusions

The PI measured using consumer-grade pulse oximeters on different fingers may provide different readings. The highest PI reading is found on the middle finger. Clinicians and primary care physicians should consider the differences in measured PI among different fingers and should use the readings with caution for any diagnostic purposes.

## Introduction

Pulse oximetry is the easiest non-invasive method to measure oxygen saturation in the blood [1]. Portable, consumer-grade, and affordable pulse oximeters are available in the market and can be used as a home health monitoring device [2]. Pulse oximeters are commonly attached to the fingertips in home and hospital settings and are sometimes attached to the ear lobe or toe. The probe of the meters contains photoplethysmograph sensors that help analyze the comparative absorption of a red and infrared wave by oxygenated and deoxygenated pulsatile blood flow [3].

In addition to measuring oxygen saturation, pulse oximeters provide a perfusion index (PI). PI is an indirect measure of peripheral perfusion status. The pulsatile signal (produced by arterial flow) is expressed as a percentage of the non-pulsatile signal (stagnant blood) to compute the PI [4]. PI is used in various clinical settings, including critical care settings, perioperative monitoring during cesarean sections, and while performing a stellate ganglion block to measure its efficacy [5-7].

A previous study by Sapra et al. reported that the highest PI is found on the right ring finger and the lowest on the right thumb among healthcare workers [8]. In contrast, Tripathy et al. showed that PI measured on different fingers varies, with the middle finger having the highest value, and the little finger having the lowest value [9]. However, to our knowledge, no study has ascertained the reliability of PI measurements on different fingers.

With this background, this study aimed to compare PI in fingers of both hands in apparently healthy individuals. The findings of this study would help find the variation and reliability of PI measured among different fingers using a pulse oximeter. According to the findings, primary care physicians can decide if the PI measured by portable oximeters can be used to detect poor perfusion.

## Materials and methods

Ethics

This study was conducted among adult (aged >18 years) research participants recruited from a tertiary care teaching hospital located in eastern India. The participants were briefed about the aims, nature, and implications of the study with an emphasis on the study procedure. After the briefing, those who provided written consent for participation were included in the study. A formal clearance from the Institutional Ethics Committee was obtained for the study (reference: HMCH/IEC/2022/160).

Type and settings

This was a cross-sectional, observational study conducted in the clinical physiology laboratory of the hospital. The laboratory was illuminated by both natural and diffuse white light. There were no direct sun rays or artificial light beams near the site where the measurements were done. The study was conducted from January to February 2022.

Minimum sample size

A study by Tripathi et al. found that there is a variation in oxygen saturation and PI in left and right-hand fingers [9]. Considering the study as a reference, we calculated the minimum sample size with the following input: α = 0.05 (p-value ≤0.05 was considered statistically significant), β = 0.1 (power of the study was 90%), and the mean PI in the right and left middle finger = 3.3 and 2.7 with an expected standard deviation of 1.7. The calculated sample size was 337 [10].

Recruitment

We obtained a convenience sample from a particular point in time (January to February 2022) from a tertiary care teaching hospital. The inclusion criteria included a declaration of apparently healthy status of the participants and providing written consent for voluntary participation by adults aged >18 years. Participants with any acute or chronic disease, taking medicine for any disease, suffering from hypertension, suffering from any vascular diseases, and suffering from any pigment disorder of fingers were excluded from the final study sample.

Measurements

All measurements were conducted between 10 am and 12 pm to avoid any potential effect of the circadian rhythm. Age was recorded in completed years as declared by the research participants. Height was measured using a portable stadiometer to the nearest 0.1 cm. Weight was measured on a digital weighing scale with an accuracy of ±0.1 kg. Waist circumference and hip circumference were measured using a fiberglass measuring tape to the nearest 0.1 cm to calculate the waist-to-hip ratio. All measurements were done by an expert clinician with experience in anthropometric measurements in the presence of a same-sex attendant in the laboratory.

**Figure 1:**
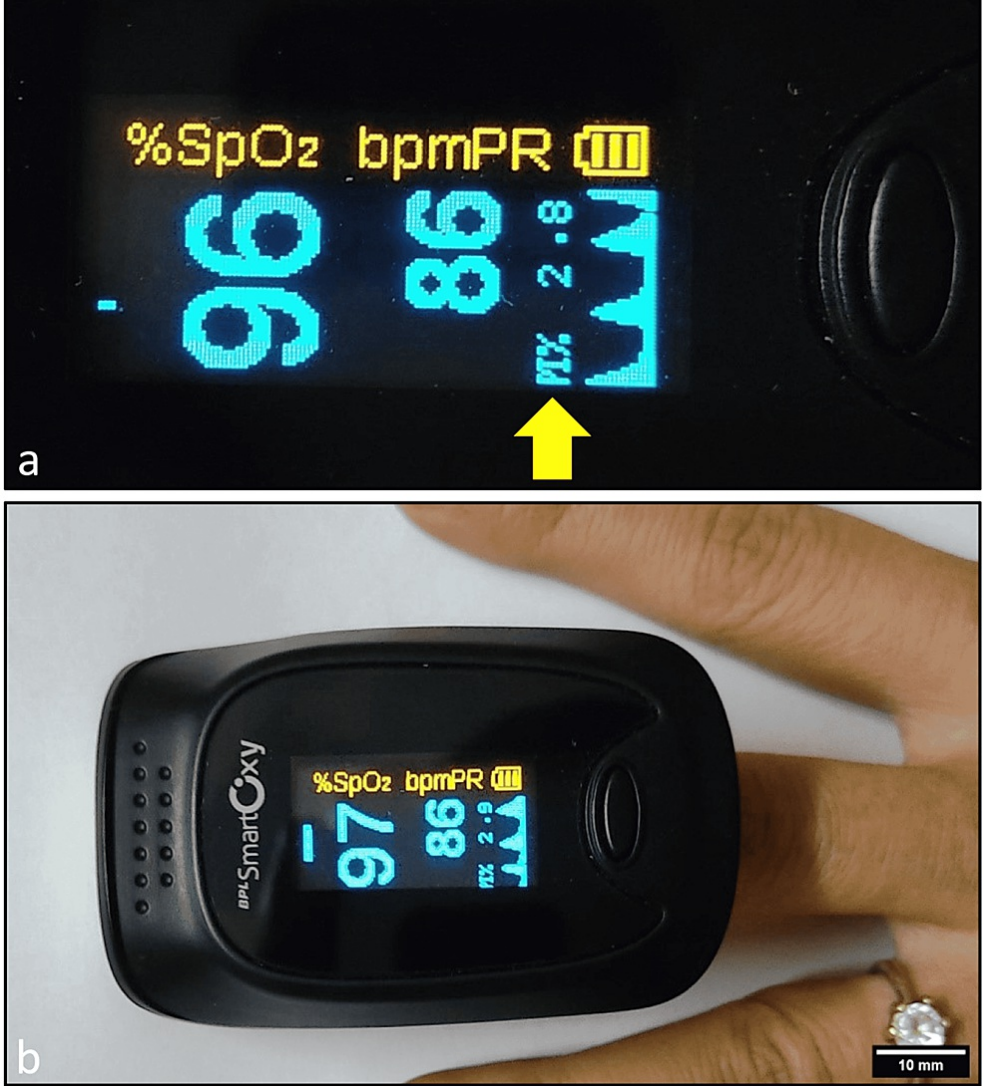
A portable pulse oximeter. (a) Screen showing a reading of heart rate, oxygen saturation, and perfusion index. (b) Measuring parameters using the oximeter on the left middle finger.

We used BPL Smart Oxy Pulse Oximeter (BPL Medical Technologies Pvt. Ltd., Bengaluru, India) for measuring the PI. Figure 1a shows a sample reading on the oximeter screen, and Figure 1b shows the probe attached to a finger.

The research participants were in a sitting posture, and the PI measurements were obtained after a five-minute rest. Although there is evidence that PI is the lowest in a sitting posture, we considered this position for participants’ convenience and the limitations of the settings [11]. Furthermore, because it is a comparative study, there would be a negligible effect of posture on the measured PI. The nails were without any color, and the fingers were without any temporary or permanent tattoo. The pulse oximeter probe was attached to the fingers one by one. After one minute of attachment on a particular finger, the reading was taken after a stable reading was seen on the screen for at least three seconds and stored for further analysis.

Statistical analysis

Data were tested for normality using the Shapiro-Wilk Test. The statistical tests were selected accordingly (normally distributed data by parametric test and non-normally distributed data by non-parametric tests) [12]. Variables between males and females were analyzed using the unpaired t-test. The variance of PI among fingers was tested by analysis of variance (ANOVA). Agreements among the measurements were tested using the intraclass correlation coefficient (ICC) model which is suitable for our measurement type [13]. The ICCs of <0.5, 0.5-0.75, 0.76-9, and >0.9 were considered “poor,” “moderate,” “good,” and “excellent” reliability, respectively [14]. The correlation coefficients ±0.0 to ±0.3, ±0.31 to ±0.5, ±0.51 to ±0.7, ±0.71 to ±0.9, and ±0.91 to ±1 were considered “negligible,” “low,” “moderate,” “high,” and “very high,” respectively [15]. The statistical analyses were carried out in GraphPad Prism 6.01 (GraphPad Software, Inc., USA) and SPSS version 20 (IBM Corp., Armonk, NY, USA). For all the tests, p-values of <0.05 were considered statistically significant.

## Results

**Figure 2:**
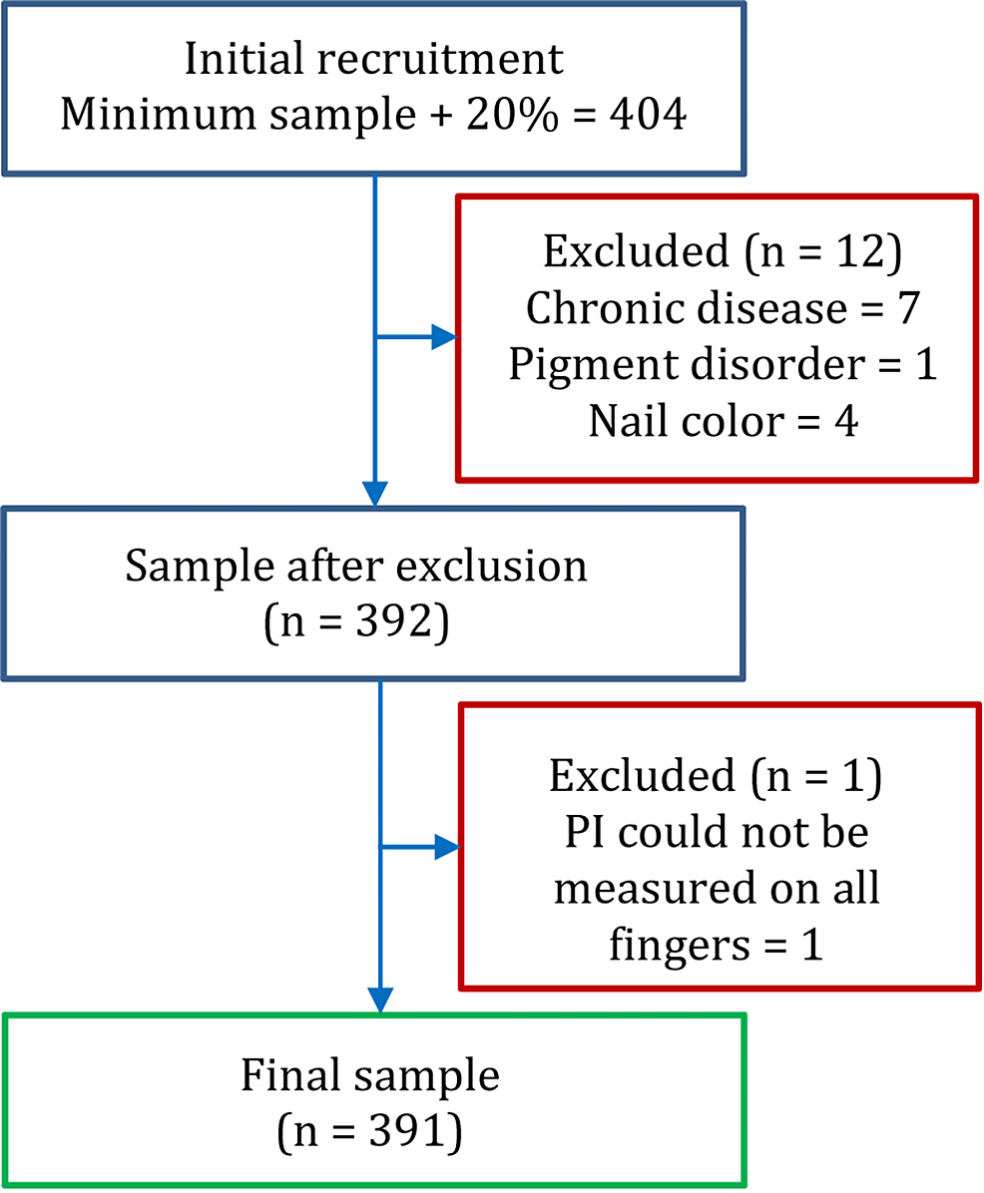
Flowchart illustrating participant recruitment. PI: perfusion index

The number of research participants initially recruited in the study and the final sample after exclusion is shown in Figure 2.

Data of 391 adult research participants with a mean age of 34.88 ± 10.65 years were analyzed. The mean age of males (n = 229 [58.57%]) was 35.1 ± 10.76 years and of females (n = 162 [41.43%]) was 34.56 ± 10.52 years (unpaired t-test, p = 0.62). The age and anthropometric variables according to gender are shown in Table 1. The height, weight, and body mass index (BMI) were higher among male participants.

**Table 1:** Overall and sex-wise age and anthropometric parameters of the participants. *Statistically significant p-values using the unpaired t-test.

Variable	Overall (n = 391)	Male (n = 229)	Female (n = 162)	P-value
Age (years)	34.88 ± 10.65	35.1 ± 10.76	34.56 ± 10.52	0.62
Height (cm)	152.19 ± 10.52	154.47 ± 12.43	148.97 ± 5.6	<0.0001*
Weight (kg)	61.97 ± 9.04	65.74 ± 8.09	56.64 ± 7.49	<0.0001*
Body mass index (kg/m^2^)	27.02 ± 4.89	27.98 ± 5.17	25.66 ± 4.13	<0.0001*
Waist-to-hip ratio	0.85 ± 0.4	0.85 ± 0.04	0.84 ± 0.05	0.06

Descriptive statistics of the measured PI on different fingers are shown in Table 2.

**Table 2:** Descriptive statistics of measured perfusion index on 10 fingers using a portable pulse oximeter in the sample (n = 391). R: right-hand fingers; L: left-hand fingers; 1-5: finger number from the thumb to the little finger; CI: confidence interval

Statistics	R1	R2	R3	R4	R5	L1	L2	L3	L4	L5
Mean	3.98	3.57	4.66	3.45	3.5	3.94	3.61	4.37	3.79	3.54
Standard deviation	1.91	1.89	2.18	1.65	1.78	1.99	2.11	2.07	2.32	2.3
Minimum	0.9	0.8	0.9	0.8	0.8	0.8	0.8	0.8	0.8	0.8
25% percentile	2.2	2.1	3	2.2	2.1	2.6	1.7	3	2.2	2
Median	3.9	4	4.6	3.3	3.1	3.4	3	4	3	3.1
75% percentile	5.4	5	5.9	4.6	5	5	5.1	5.4	5	4.4
Maximum	8.4	8.4	12.6	9.8	7	12.8	7	8.9	9.2	10
Standard error of mean	0.09	0.09	0.11	0.08	0.09	0.1	0.11	0.1	0.12	0.12
Lower 95% CI	3.79	3.38	4.44	3.29	3.32	3.74	3.39	4.17	3.56	3.32
Upper 95% CI	4.17	3.75	4.88	3.62	3.68	4.13	3.82	4.58	4.02	3.77

**Figure 3:**
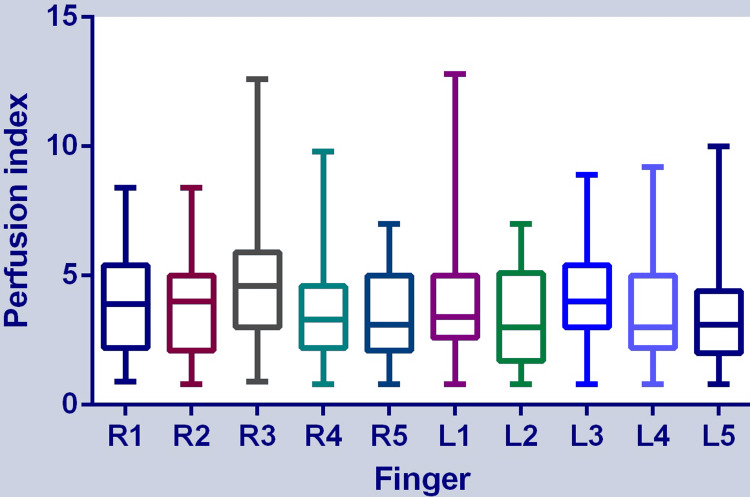
Perfusion index measured on 10 fingers using a portable pulse oximeter. “R” indicates right and “L” indicates left, and the number from 1 to 5 indicates thumb to the little finger. Repeated-measures ANOVA result: F (9, 3900) = 15.49, p < 0.0001. ANOVA: analysis of variance

The PI in the order of the right thumb to the little finger was 3.98 ± 1.91, 3.57 ± 1.89, 4.66 ± 2.18, 3.45 ± 1.65, and 3.5 ± 1.78, respectively. The PI in the order of the left thumb to the little finger was 3.94 ± 1.99, 3.61 ± 2.11, 4.37 ± 2.07, 3.79 ± 2.32, and 3.54 ± 2.3, respectively. The highest PI was found on the middle finger of both hands. There was a significant difference among the PI measured on different fingers (F (9, 3900) = 15.49, p <0.0001) (repeated-measures ANOVA) (Figure 3).

**Figure 4:**
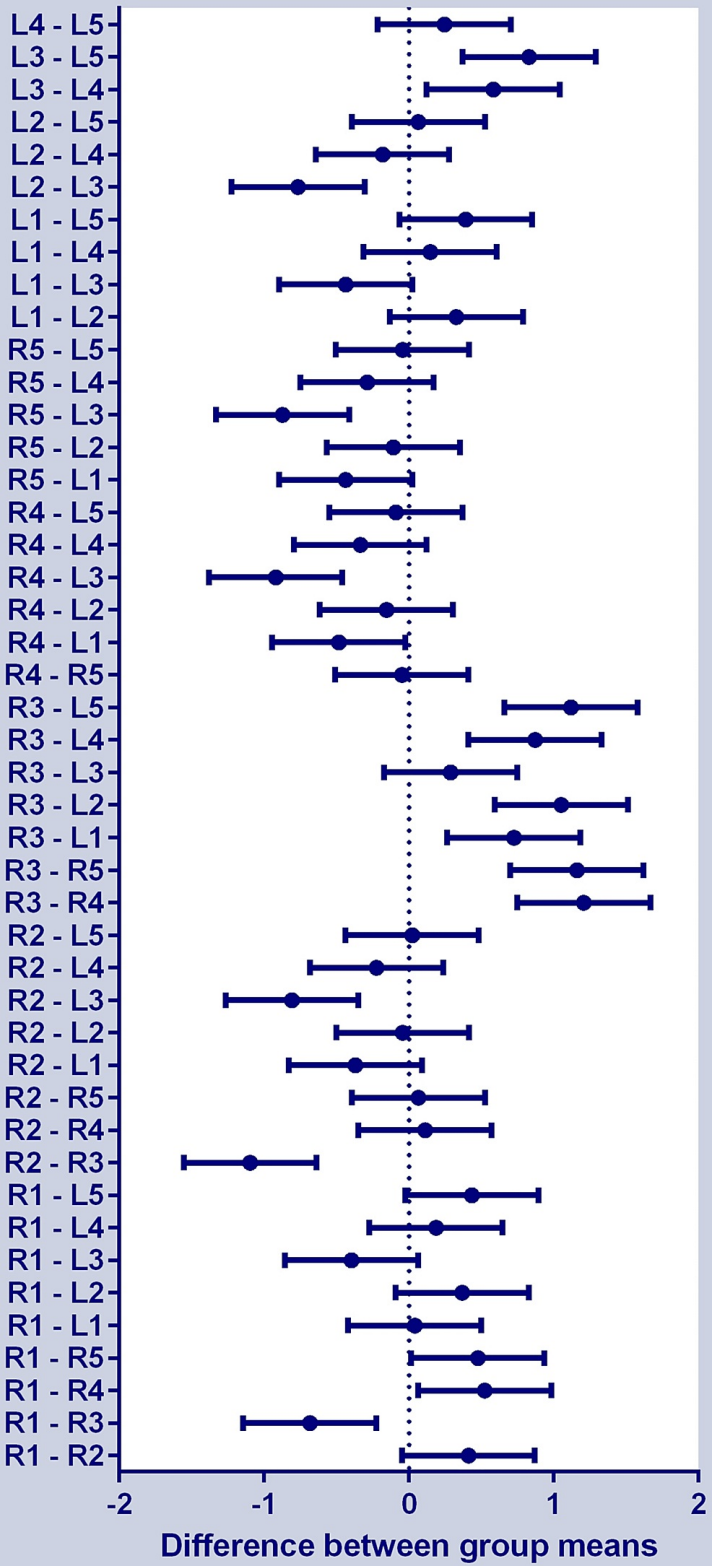
Difference between group means in Tukey’s post-hoc test among perfusion index measured in different fingers. Bar touching the zero line indicates a non-significant difference (there were 17 significant and 28 non-significant group differences). “R” indicates right and “L” indicates left, and the number from 1 to 5 indicates thumb to the little finger.

According to Tukey’s post-hoc test, there were 17 significant and 28 non-significant group differences, as shown in Figure 4.

The inter-item Pearson correlation coefficients of PI measured on 10 fingers are shown in Table 3. All items showed a statistically significant positive correlation between the pairs. The coefficients ranged from (R3 versus L2) 0.133 to (L4 versus L5) 0.798.

**Table 3:** Inter-item correlation matrix of perfusion index measured on 10 fingers among the 391 research participants. R1-R5: right thumb to right little finger; L1-L5: left thumb to left little finger. All correlation coefficients were statistically significant. Interpretation of correlation coefficient: ±0.0 to ±0.3, ±0.31 to ±0.5, ±0.51 to ±0.7, ±0.71 to ±0.9, and ±0.91 to ±1 considered to be “negligible,” “low,” “moderate,” “high,” and “very high,” respectively.

	R1	R2	R3	R4	R5	L1	L2	L3	L4	L5
R1	1.000									
R2	0.503	1.000								
R3	0.464	0.377	1.000							
R4	0.223	0.430	0.496	1.000						
R5	0.289	0.581	0.185	0.442	1.000					
L1	0.521	0.620	0.510	0.611	0.551	1.000				
L2	0.380	0.581	0.133	0.488	0.669	0.701	1.000			
L3	0.420	0.393	0.287	0.443	0.470	0.592	0.648	1.000		
L4	0.432	0.549	0.198	0.536	0.504	0.587	0.703	0.652	1.000	
L5	0.485	0.671	0.288	0.454	0.564	0.520	0.688	0.604	0.798	1.000

**Figure 5:**
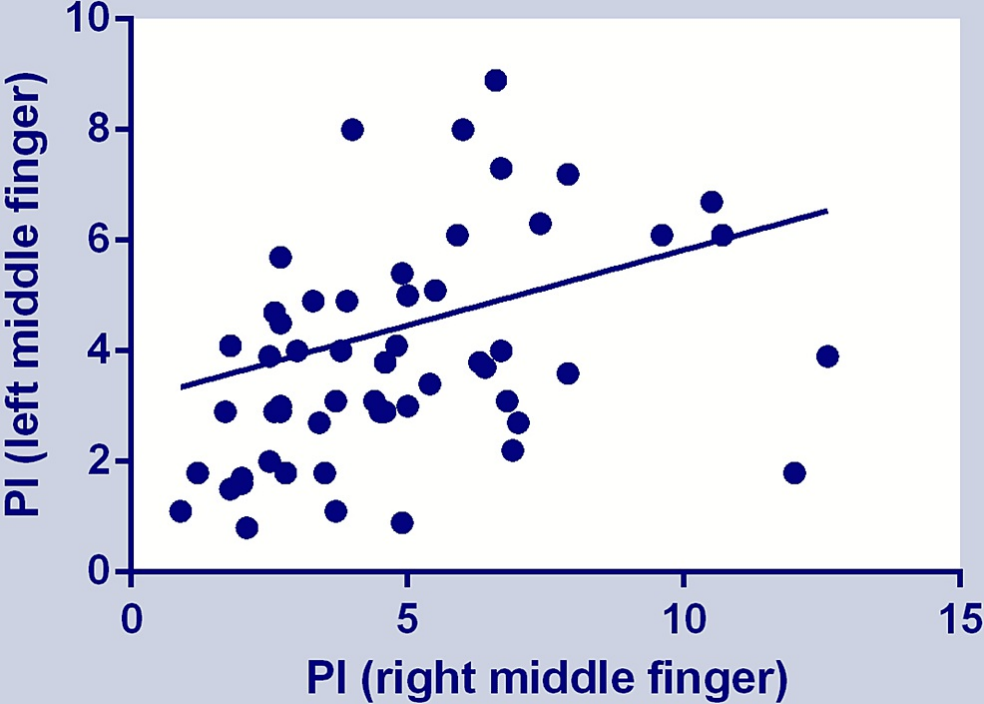
Scatter plot of PI of the left and right middle finger with the trendline. PI: perfusion index

The correlation between the left and right middle finger was 0.287 (CI = 0.193 to 0.375; p < 0.0001). The correlation with the trend line is shown in a scatterplot in Figure 5.

The ICC was 0.474, 0.368, and 0.635 for overall, right-hand, and left-hand fingers, respectively (Table 4), indicating “poor,” “poor,” and “moderate” reliability, respectively, among the measurements.

**Table 4:** Intraclass correlation coefficients of overall, right, and left-hand finger perfusion index measurement. According to the data, ICC model 3 was used with SPSS input as a “two-way mixed” model and “absolute agreement” type. ICC: intraclass correlation coefficient; CI: confidence interval

	ICC (single measure)	95% CI		F test with true value 0			
		Lower bound	Upper bound	Value	df1	df2	P
Overall	0.474	0.431	0.518	10.688	390	3510	<0.0001
Right	0.368	0.310	0.427	4.252	390	1650	<0.0001
Left	0.635	0.59	0.679	10.262	390	1650	<0.0001

## Discussion

Regarding the agreement among PI measured on 10 fingers, we found a significant difference among the measurements. The middle finger showed the highest measured PI among the fingers of a hand. Although the correlation between the PI of the left and right middle finger was statistically significant, the coefficient of determination (r^2^) shows that approximately 8% of the variation in PI from the left middle finger can be predicted from the right middle finger, or vice versa [16].

The PI is calculated from the photoplethysmographic signals by comparing the pulsatile to non-pulsatile peripheral circulation. Two principal factors may influence PI, namely, cardiac output and the balance between the sympathetic and parasympathetic nervous systems.

If there is higher cardiac output and/or parasympathetic predominance, there is higher PI. In contrast, when the cardiac output is lower and/or sympathetic predominance, there is low PI. The normal range of PI is considered to be between 0.2% and 20% [17]. In our study, we found the PI in the left middle finger to be 4.37 ± 2.07% and the right middle finger to be 4.66 ± 2.18%. However, we did not measure the cardiac output and autonomic nervous system to comment on the associated cardiac and nervous system status. Our findings corroborate those of Savastan et al. who found the PI to be 4.3 (interquartile range = 2.9-6.2) among apparently healthy subjects with a median age of 42 (interquartile range = 33-47) years [18].

At the physiological state, posture can affect the measured PI, and it is found that it is the lowest in the sitting posture and the highest in the Trendelenburg position [11]. In our study, we used the sitting posture in all participants. Hence, we presume that the PI was at the lowest level when we compared it on the fingers. As the PI is the ratio of pulsatile to non-pulsatile blood flow, any disease that compromises the blood flow to the periphery would affect the PI [19]. The PI in emergency departments helps in the identification of the need for transfusion [20]. It also helps to estimate the mortality risk in patients presenting with upper gastrointestinal bleeding and mortality in mechanically ventilated patients [21,22]. Moreover, it helps in the detection of hypotension during anesthesia and to identify the effectiveness of ganglion blocks [6,7]. In intensive care settings, pulse oximetry is an integral part of monitoring other parameters in single hospital-grade devices. However, we used a consumer-grade device in this study. Hence, the results of this study may not be compared with studies where hospital-grade oximeters were used.

Primary care physicians and general physicians may use consumer-grade pulse oximeters for home visits for measuring the oxygen saturation (SpO_2_) of patients and PI. They should take precautions to minimize the patient-to-patient transmission of disease by using probes or finger covers [23]. However, they should be cautious that PI measured on different fingers may show different readings. Previous studies have established that the middle fingers show higher SpO_2_ levels when compared with other fingers [24,25]. In this study, we found that the PI also shows the highest reading on the middle finger in both limbs.

Limitations

This study has some limitations. We recruited the study sample from a hospital. The convenience sample is a non-probability sample. Hence, it is not possible to estimate how well it represents the population. In addition, we only recruited the sample from apparently healthy individuals to determine PI in normal physiology. Hence, the study findings may not be extended to people with any particular diseases.

## Conclusions

The PI measured by portable and consumer-grade pulse oximeters on different fingers of the left and right hand may be different. The highest reading of PI is obtained on the middle finger in each hand. There is poor reliability of measured PI on different fingers by a pulse oximeter. Hence, the PI obtained from oximeters should be interpreted with caution for any diagnostic purposes. Further studies need to be conducted to compare the reliability of hospital-grade and consumer-grade oximeters in the measurement of PI on different fingers.
